# Endotracheal Intubation in Early Life and Its Association with Mental Health Disorders from Childhood to Adolescence: A National Longitudinal Cohort Study up to Age 18

**DOI:** 10.3390/jcm15020824

**Published:** 2026-01-20

**Authors:** Ga Won Jeon, Nayoung Jung, Jaeho Shin, Ju Hee Kim, Eunkyo Ha, Seonkyeong Rhie, Man Yong Han

**Affiliations:** 1 Department of Pediatrics, Kangbuk Samsung Hospital, Sungkyunkwan University School of Medicine, Seoul 03181, Republic of Korea; iamgawon@hanmail.net; 2 Department of Pediatrics, Chung-Ang University Gwangmyeong Hospital, Gwangmyeong 14353, Republic of Korea; 119youngna@gmail.com; 3 Department of Surgery, Incheon St. Mary’s Hospital, Incheon 21431, Republic of Korea; xenic@hanmail.net; 4 Department of Pediatrics, Kyung Hee University Medical Center, Kyung Hee University College of Medicine, Seoul 02447, Republic of Korea; 2004052@gmail.com; 5 Department of Pediatrics, Hallym University Kangnam Sacred Heart Hospital, Seoul 07441, Republic of Korea; dmsry1@gmail.com; 6 Department of Pediatrics, CHA Bundang Medical Center, CHA University School of Medicine, Seongnam 13496, Republic of Korea

**Keywords:** intratracheal intubation, mental disorders, neonatal intensive care, cohort studies

## Abstract

**Background/Objectives:** The long-term impact of intubation during infancy or early childhood on later childhood or adolescence remains unclear. This study investigates the association between early-life intubation and subsequent mental health outcomes. **Methods:** We conducted a retrospective cohort study using nationwide data on children born in Korea between 2002 and 2005. Those who underwent intubation (exposed cohort) were compared with 1:10 matched unexposed controls who did not undergo intubation. **Results:** The exposed cohort (*n* = 18,799) had a significantly higher incidence rate of mental health disorders than controls (28.2 vs. 13.9 per 1000 person-years; HR 1.82, 95% CI 1.74–1.93). Autism spectrum disorder (HR 3.09) and attention-deficit/hyperactivity disorder (HR 1.61) increased in early childhood, while bipolar disorders (HR 2.36), schizophrenia spectrum disorders (HR 2.27), depressive disorders (HR 1.94) and anxiety disorders (HR 1.84) increased in adolescence. Higher incidence was noted in females, children not admitted to intensive care units, and those without congenital heart disease or bronchopulmonary dysplasia (*p* < 0.05). Hospitalization length correlated with mental health outcomes (*p* < 0.001), but ventilator duration did not (*p* = 0.694). **Conclusions:** Early-life intubation is associated with an increased risk of mental health disorders, highlighting the need for long-term follow-up and support for these children. In particular, increased clinical awareness is needed during follow-up care for patients at higher risk, such as females, children without congenital heart disease or bronchopulmonary dysplasia, those intubated at an older age, and those with longer hospitalizations.

## 1. Introduction

Endotracheal intubation is a common medical procedure, particularly in preterm infants or those with respiratory distress. It is vital during critical moments of life and can be lifesaving [1]. However, while essential for immediate survival, endotracheal intubation may contribute to anxiety, post-traumatic stress disorder, and emotional distress in early life, potentially disrupting neurodevelopmental processes [2]. Recent evidence also raises concerns about its potential long-term effects, including cognitive, emotional, and behavioral challenges later in childhood and adolescence [3,4,5]. Research on the long-term mental health effects of endotracheal intubation, however, remains limited [6]. While short-term neurodevelopmental outcomes, such as impaired brainstem development and abnormal white matter maturation in preterm infants, are well-documented [5], existing studies primarily focus on early-onset conditions such as autism spectrum disorder (ASD) and attention-deficit/hyperactivity disorder (ADHD) [6,7]. For instance, prolonged mechanical ventilation in preterm infants has been associated with increased risks of ASD [8], intellectual disability [1], and ADHD [6]. However, these studies overlook broader child and adolescent mental health outcomes and the long-term implications of intubation beyond early childhood. Additionally, factors such as the timing and duration of intubation, as well as stress from invasive procedures and intensive care environments, remain underexplored [2,9]. Addressing these gaps is critical for understanding how intubation influences neurodevelopment and mental health trajectories, ultimately guiding interventions that support both immediate survival and long-term well-being.

This study investigates the long-term association between endotracheal intubation in infancy or early childhood and subsequent mental health disorders using national cohort data from birth to 18 years. We aim to determine whether early-life intubation is a significant risk factor and to identify factors that influence its impact, ultimately informing early interventions, monitoring, and targeted support for at-risk children

## 2. Materials and Methods

### 2.1. Study Design

This retrospective, population-based cohort study included individuals born in South Korea between 2002 and 2005, as identified through the National Health Insurance Service (NHIS) database and linked to census data from Statistics Korea [10]. The NHIS provides healthcare coverage for over 98% of the South Korean population and contains claims-based medical data. The dataset included information on demographic characteristics, healthcare utilization (including diagnostic codes from the 10th revision of the International Classification of Diseases, ICD-10) [11], prescriptions, and relevant procedures. Intubation and ventilation procedure codes were extracted from the ICD using reimbursement codes from the National Health Insurance procedure classification. Participants were followed until the first diagnosis of a mental health disorder, death, or the end of the study (31 December 2021), whichever occurred first.

### 2.2. Study Population

**Figure 1:**
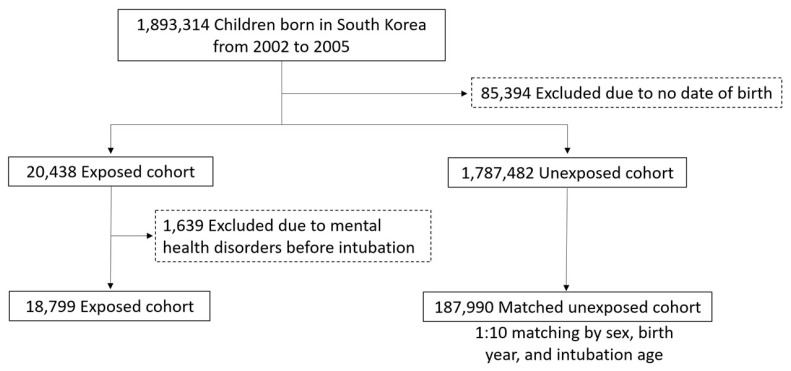
Study population. A total of 1,893,314 individuals born in South Korea between 2002 and 2005 were identified from the National Health Insurance Service database. We excluded 85,394 individuals due to missing birth date information. Children who underwent intubation were assigned to the exposed cohort. We further excluded 1639 children diagnosed with a mental health disorder prior to intubation, leaving 18,799 individuals in the exposed cohort. A 1:10 matched unexposed cohort was selected using random matching based on sex, birth year, and age at intubation. The final study population consisted of 18,799 children in the exposed cohort and 187,990 in the matched unexposed cohort.

This cohort study included 1,893,314 individuals born in South Korea between 2002 and 2005 who were followed from birth until 31 December 2021 (Figure 1). We excluded 85,394 individuals due to missing birth date information. Children who underwent intubation were assigned to the exposed cohort, while those who did not were assigned to the unexposed cohort and matched to the same intubation age as their exposed counterparts. In the unexposed cohort, follow-up continued until a mental health diagnosis or the occurrence of intubation. We further excluded 1639 children diagnosed with a mental health disorder prior to intubation, leaving 18,799 individuals in the exposed cohort (i.e., those with a T30 prescription code). A 1:10 matched unexposed cohort was then selected using random matching based on sex, birth year, and intubation age. The final study population consisted of 18,799 children in the exposed cohort and 187,990 in the matched unexposed cohort.

### 2.3. Exposure

The exposed cohort included individuals who underwent intubation during hospitalization, regardless of the reason, within the follow-up period. The M5859 code was primarily used to identify cases of endotracheal intubation, while intubations performed via cricothyrotomy were identified using the M583 code. The duration of ventilator use was also extracted from procedure codes, with the M58 code commonly used for this purpose. Intubations conducted using high-frequency oscillatory ventilation modes were identified using the M08 code. Both M58 and M08 codes include subcategories classifying ventilation duration as follows: within 3 h (M50), 3–8 h (M57), 8–12 h (M58), and over 12 h (M60). For cases exceeding 12 h, ventilation duration was calculated as one day, allowing precise quantification of intubation durations. In our study, intubation was defined as a binary exposure based on the first occurrence of endotracheal intubation in early life. Children who experienced more than one intubation episode were excluded from the study population at baseline. Repeated intubation episodes are likely to reflect a substantially different clinical trajectory, including greater underlying disease severity and prolonged exposure to intensive care-related factors, which could introduce additional heterogeneity and confounding. By restricting the exposure definition to the first intubation event and excluding children with repeated episodes, we aimed to improve cohort homogeneity and reduce potential misclassification and residual confounding.

### 2.4. Mental Health Disorders

We analyzed 24 mental health disorders using the Child and Adolescent Mental Health Disorders Classification System, which aligns with the diagnostic groups defined in the Diagnostic and Statistical Manual of Mental Disorders, Fifth Edition (DSM-5). Data were extracted using ICD-10 codes (Table 1) [11]. Medical resource utilization, including inpatient and outpatient visits, was recorded throughout the follow-up period.

### 2.5. Covariates

Adjustments were made for various confounders, including sex, socioeconomic status, birth residence, and perinatal status. Intensive care unit (ICU) admission status was extracted from procedure codes during the follow-up period. Bronchopulmonary dysplasia (BPD) was defined according to the 2001 National Institute of Child Health and Human Development consensus definition. Respiratory distress syndrome was identified using the diagnostic code P22X, and congenital heart disease (CHD) was identified based on diagnostic codes Q20X-Q28X. Socioeconomic status was categorized as low (<25th percentile), intermediate (25th–75th percentile), or high (>75th percentile) based on medical fees. Birth residence was classified as Seoul, metropolitan areas, cities or rural regions, with missing data categorized separately. Perinatal status was constructed to reflect the overall presence of early-life perinatal adversity or biological vulnerability at birth as a potential confounder.

### 2.6. Statistical Analysis

We first calculated the number and percentage of the 24 mental health disorders in both the exposed and matched unexposed cohorts, as well as the median age (interquartile range, IQR) at the time of diagnosis. Incidence rates were calculated as new cases per 1000 person-years over the 18-year observation period. Conditional Cox models were employed to estimate hazard ratios (HRs) with 95% confidence intervals (CIs) for the exposed cohort, assessing risk factors for 24 mental health disorders associated with endotracheal intubation from the index date onward. Mental health disorders with fewer than 50 cases were not analyzed separately but were included in the overall HR calculations. Sexuality and gender identity (7 cases), personality disorder (23 cases), and dissociative disorder (2 cases) were not analyzed separately, but were included in the “any disorder” count.

The Cox proportional hazards model was validated using log-minus-log plots and Schoenfeld residuals, with covariates categorized as previously described. Confounding variables included sex, birth year (already matched), socioeconomic status (low, intermediate, and high), birth residence (Seoul, metropolitan, cities, and rural regions), ICU admission within 3 months (yes vs. no), and perinatal status (yes vs. no). In the stratified analysis, HRs were estimated by sex, calendar year at birth, birth residence, ICU admission within 3 months of birth, any perinatal status, age at intubation (<4 month vs. 4–23 month vs. >23 month), respiratory distress syndrome, BPD, and CHD, with interaction *p*-values calculated to assess heterogeneity between subgroups. The dose–response analysis, based on the duration of hospital stay (≤25 vs. >25 days) and ventilator duration (≤12 vs. >12 days), was conducted alongside statistical comparisons against the control cohort. *p*-values for the comparison of HRs between subgroups were calculated using the difference in log HRs with a Wald test.

Sensitivity analyses were performed using more stringent definitions of mental health conditions, including cases with >2, >3, or >5 disorders, as well as an alternative outcome definition. Inverse probability of treatment weighting (IPTW) was used to adjust for confounding by assigning weights based on the inverse of the propensity score for sensitivity analysis [12]. Propensity scores were estimated using logistic regression, and the weights were used to create a balanced pseudo-population, ensuring treatment assignment was independent of observed covariates [12]. Statistical analyses were conducted using SAS version 9.4 (SAS Institute Inc., Cary, NC, USA), and a two-sided *p*-value < 0.05 was considered statistically significant.

## 3. Results

### 3.1. Baseline Characteristics of the Participants

Among the 1,893,314 individuals, intubation was performed on 20,438 participants (0.01%) during the study period. After excluding individuals with prior diagnoses of mental health disorders before the index date, 18,799 participants were assigned to the exposed cohort, and 187,990 participants were included in the randomly exact-matched 1:10 unexposed cohort. The median age (IQR) at the index date was 3.0 months (0–21 months) in the exposed cohort. The two cohorts were identical regarding the matching criteria, including sex (male: 56.8%) and birth year (2002–2003: 50.3%) (Table 2). The cohorts also showed similar distributions of birth residence and socioeconomic status. Compared with the unexposed cohort, the exposed cohort had a higher prevalence of perinatal status (76.1% vs. 24.0%), BPD (3.2% vs. 0.01%), respiratory distress syndrome (24.4% vs. 0.3%), and CHD (44.5% vs. 1.8%). Details of the perinatal status are provided in Table 3.

### 3.2. Mental Health Disorders: Incidence, HRs, and Age at Diagnosis

**Figure 2:**
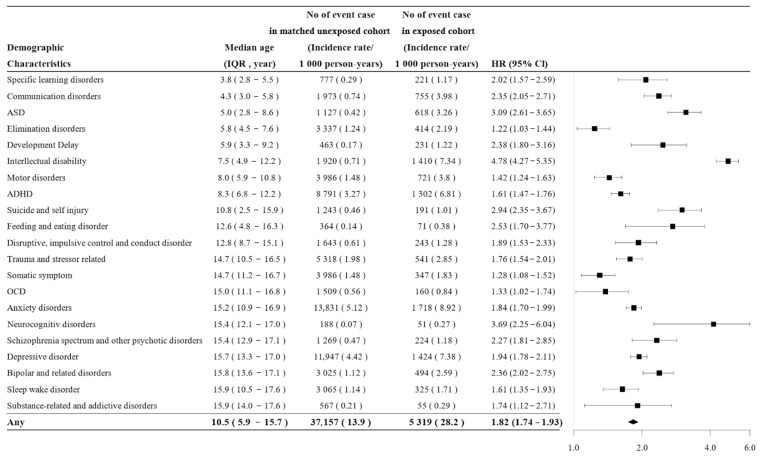
Mental health disorders: incidence, hazard ratios, and age at diagnosis. The incidence of mental health disorders was 28.2 vs. 13.9 cases per 1000 person-years in exposed and unexposed cohorts, respectively. Exposed individuals had higher rates in 21 of the 24 disorders, most commonly anxiety (8.92 vs. 5.12), depression (7.38 vs. 4.42), intellectual disability (7.34 vs. 0.71), and ADHD (6.81 vs. 3.27). Overall risk was 1.82-fold higher, with the greatest risk for intellectual disability (HR 4.78), ASD (HR 3.09), neurocognitive disorders (HR 3.69), suicide and self-injury (HR 2.94), and feeding and eating disorders (HR 2.53). The median age at diagnosis of mental health disorders was 10.5 years (ASD, 5 years; ADHD, 8.3 years; anxiety disorders, 15.2 years; depressive disorders, 15.7 years). ASD, autism spectrum disorder; ADHD, attention-deficit/hyperactivity disorder; OCD, obsessive–compulsive disorder; HR, hazard ratio; CI, confidence interval.

The incidence rate of mental health disorders was 28.2 per 1000 person-years (5319 cases) in the exposed cohort and 13.9 per 1000 person-years (37,157 cases) in the unexposed cohort. The exposed cohort had a 1.82-fold higher risk for mental health disorders compared with the unexposed cohort (HR 1.82, 95% CI 1.74–1.93). The median age at diagnosis was 10.5 years (IQR: 5.9–15.7) (Figure 2). Exposed individuals had higher rates in 21 of the 24 mental health disorders (excluding three disorders with fewer than 50 cases), most commonly anxiety disorders (8.92 vs. 5.12 per 1000 person-years), depressive disorders (7.38 vs. 4.42), intellectual disability (7.34 vs. 0.71), and ADHD (6.81 vs. 3.27). The exposed cohort also had higher risk in 21 of the 24 mental health disorders (excluding three with fewer than 50 cases), with the greatest risk for intellectual disability (HR 4.78), ASD (HR 3.09), neurocognitive disorders (HR 3.69), suicide and self-injury (HR 2.94), feeding and eating disorders (HR 2.53), bipolar disorders (HR 2.36), schizophrenia spectrum disorders (HR 2.27), depressive disorders (HR 1.94), and anxiety disorders (HR 1.84). The median age at diagnosis was 5 years for ASD and 8.3 years for ADHD, both typically early childhood disorders, and 15.2 years for anxiety disorders and 15.7 years for depressive disorders, typically diagnosed in late childhood or adolescence.

### 3.3. Stratified Analysis

The incidence rate of mental health disorders was adjusted for multiple confounders through stratification (Table 4). Stratified analysis revealed significant interactions between mental health disorders and sex, age at intubation, perinatal status, and CHD. Females had a higher risk (HR 1.93, 95% CI 1.80–2.07) than males (HR 1.74, 95% CI 1.64–1.85) (*p* = 0.008). In the exposed cohort, incidence rates were higher than in the control group for both sexes. These findings suggest that intubation increases the risk of mental health disorders in both sexes, with a relatively greater impact observed in females. The interaction was also observed across age groups, with risk increasing with age at intubation: HR 1.34 (95% CI 1.17–1.54) for <4 months, 1.47 (95% CI 1.37–1.57) for 4–23 months, and 2.45 (95% CI 2.27–2.64) for ≥24 months (*p* < 0.001). Individuals without perinatal complications (HR 3.06, 95% CI 2.81–3.32) or CHD (HR 2.68, 95% CI 2.52–2.85) had higher risks than those with perinatal complications (HR 1.55, 95% CI 1.47–1.63) or CHD (HR 1.30, 95% CI 1.19–1.42) (each *p* < 0.001).

### 3.4. Dose–Response Analysis

The median length of hospitalization at the time of intubation was 25 days (IQR: 15–40 days). In the dose–response analysis, children hospitalized for ≤25 days had an incidence of 22.6 per 1000 person-years (HR 1.68, 95% CI 1.60–1.77), whereas those hospitalized for >25 days had 33.5 per 1000 person-years (HR 2.77, 95% CI 2.62–2.93; *p* < 0.001), indicating a significantly higher risk. The median ventilator duration was 12 days (IQR: 11–15 days), and there was no significant difference in mental health risk by ventilator duration (HR 1.89 vs. 2.02; *p* = 0.694) (Table 5).

### 3.5. Sensitivity Analysis

The incidence rate of multiple mental health disorders was higher in the exposed cohort compared to the unexposed cohort. Among individuals with more than two mental health disorders, the incidence rate was 146.3 versus 58.6 per 1000 person-years in the exposed and unexposed cohorts (HR 2.51, 95% CI 2.41–2.62). For those with more than three disorders, the incidence rates were 81.2 versus 31.1 (HR 2.63, 95% CI 2.49–2.78), and for more than five disorders, 25.2 versus 8.9 (HR 2.82, 95% CI 2.55–3.11) (Table 6). Sensitivity analysis using IPTW produced similar results (Table 7).

## 4. Discussion

In this study, we identified a significant association between intubation during infancy or early childhood and an increased risk of mental health disorders before age 18. Notably, the risk was higher in females, children without BPD or CHD, those intubated at an older age, and those with longer hospitalization. However, ventilator duration was not directly associated with increased risk. Our analysis extends previous research by examining not only mental health issues in early childhood but also a broader range of disorders, including those more commonly diagnosed in adolescence.

We speculate that the impact of intubation on long-term mental health outcomes is likely due to the physiological and environmental effects of the procedure itself rather than the underlying conditions that necessitated it [13,14]. Severe medical conditions, such as CHD or BPD, contribute to neurodevelopmental impairments [15]; however, growing evidence suggests that intubation and its associated interventions independently influence mental health outcomes. Two primary mechanisms may explain this association. First, neonatal intubation may alter neurodevelopmental pathways through hypoxia, oxidative stress, and neuroinflammation [16,17,18,19]. These processes can disrupt white matter integrity and synaptic connections [20], thereby increasing the risk of cognitive and emotional disorders such as ADHD and ASD. Second, the critical care environment itself may induce chronic stress [21]. Prolonged hospitalization, separation from caregivers, and repeated invasive procedures may dysregulate the hypothalamic–pituitary–adrenal axis, heightening susceptibility to anxiety and mood disorders [13]. In the present study, ventilator duration was not associated with the risk of mental health disorders, whereas longer hospital stays were linked to an increased risk. This suggests that factors related to the care environment, rather than intubation itself, may contribute to these mental health outcomes. However, this finding may also reflect the possibility that ventilator duration was misclassified or insufficiently granular.

Despite such research, it remains unclear whether the observed mental health outcomes are directly attributable to the intubation procedure itself or are a consequence of the underlying medical conditions necessitating it. This uncertainty arises because preterm infants or those with significant perinatal complications, such as BPD or CHD, are typically considered at higher risk for neurodevelopmental issues [22,23,24,25,26]. In animal models of hyperoxia-induced lung injury resembling BPD, hyperoxia-activated circulating extracellular vesicles are thought to mediate both lung and brain injury, suggesting a link between lung injury and neurodevelopmental impairment in infants with BPD [27]. Neurodevelopmental impairments are also a common outcome among hospitalized neonates with CHD requiring critical care [28]. However, we found that the risk of developing mental health disorders was higher in the absence of these conditions. The higher risk of mental health disorders in infants without BPD or CHD may reflect the routine neurodevelopmental follow-up and early intervention provided for infants with these conditions in Korea. It may also indicate that intubation itself, rather than the underlying medical conditions, contributes to adverse mental health outcomes. Similarly, in our study, BPD was not associated with an increased risk of ADHD in adulthood [29]

The exact mechanism remains unclear; however, disorders that manifest in late childhood or adolescence, such as anxiety or depression, are presumed to be related to hypoxia or stress-related pathways [30]. Our findings indicate that the risk of mental health disorders increased with hospital stays longer than 25 days, reinforcing the impact of prolonged hospitalization on mental health outcomes. Prolonged hospitalization reduces opportunities for critical social interaction during brain development and leads to separation from caregivers, both of which may adversely affect neurodevelopment. Such reductions in social interaction and caregiver contact are thought to have lasting effects into late childhood and adolescence [31]. The absence of essential mother–child interaction during early childhood—a period when brain development is highly sensitive to environmental influences—due to prolonged hospital stays is associated with impaired emotional development and poor mental health outcomes [32,33]. In addition, chronic hypoxia from prolonged intubation may induce sustained neuroinflammation that disrupts brain function, thereby increasing the risk of anxiety, depression, or neurodevelopmental disorders in children [34].

The strengths of this study are as follows. First, it is based on a nationwide cohort with a large sample size of 1,893,314 participants, which enhances the reliability and generalizability of the findings. Second, the study comprehensively addresses a wide range of mental health disorders, from those diagnosed in early childhood to late childhood and adolescence, allowing for a thorough analysis across different age groups. Additionally, stratified, dose–response, and sensitivity analyses were conducted to examine robust associations between various factors and mental health disorders. However, there are some limitations. First, the data used in this study were primarily derived from the NHIS and classified based on recorded codes, which may not fully capture the accurate diagnosis and management of all mental health disorders, as some conditions may have been diagnosed later or missed. Second, information on sedative, analgesic, and anesthetic exposure was not included in the data request and therefore could not be incorporated as confounders in the analysis. These medications are closely linked to disease severity and intensive care interventions and may function as intermediate variables on the causal pathway or introduce the risk of overadjustment. Third, perinatal status represents diverse pathophysiological processes. It reflects the overall presence of early-life perinatal adversity or biological vulnerability as a potential confounder, rather than estimating the independent effect of each individual condition. In large claims-based datasets, perinatal diagnoses are often rare, overlapping, and lack severity detail, making separate adjustment impractical and unstable. Using a binary composite indicator allowed us to adjust for broad perinatal risk while preserving model stability and minimizing overfitting, but it may mask condition-specific heterogeneity and prevent assessment of individual neurodevelopmental risks. Fourth, the cutoffs used for length of hospital stay and ventilator duration were data-driven and based on the median values within the study population (25 days vs. 12 days, respectively). Given the lack of universally accepted clinical thresholds for defining “prolonged” hospitalization or ventilator use in this heterogeneous pediatric population, the median-based approach was chosen to ensure balanced group sizes and statistical stability, particularly in claims-based data with skewed distributions. Alternative approaches—quartile-based cutoffs, clinically defined thresholds where available, or modeling duration as a continuous variable—are also possible. However, the primary aim of this analysis was exploratory stratification rather than precise threshold determination.

## 5. Conclusions

Endotracheal intubation during infancy or early childhood is associated with an increased risk of mental health disorders in early childhood, later childhood, and adolescence, with intubated children showing a 1.82-fold higher risk than their non-intubated peers. Longer hospital stays further increase this risk. Reducing unnecessary intubations, minimizing hospital stays, and promoting early parent–infant bonding may help reduce these risks. These findings underscore the importance of recognizing intubation as an independent risk factor and highlight the need for monitoring and early intervention. In particular, clinicians should monitor these patients more closely during follow-up of higher-risk patients—females, children without BPD or CHD, those intubated at an older age, and those with longer hospital stays—and provide prompt intervention when problems are identified. Further prospective studies are required to evaluate the causal relationship between endotracheal intubation and mental health disorders, as well as to evaluate the mechanisms underlying specific mental health outcomes.

## Figures and Tables

**Table 1:** Mental health disorders and their respective ICD-10 codes.

MeSH Term	ICD-10 Code	MeSH Term	ICD-10 Code
Obsessive-compulsive disorders	F42X	Motor disorders	F95X, F82
Intellectual disability	F7X	Specific learning disorders	F801
Anxiety disorders	F40X	Autism spectrum disorders	F84X
Attention-deficit hyperactive disorders	F90X	Schizophrenia spectrum and other psychotic disorders	F060, F200X
Bipolar and related disorders	F301X	Somatic symptom	F444
Communication disorders	F80X	Trauma and stress related	F43X
Depressive disorders	F32X	Sleep–wake disorders	F 52X, F62X
Developmental delay	F8189, F819, F88, F89	Neurocognitive disorders	F0150, F063X
Disruptive impulsive control and conduct disorders	F630, F639, F91X	Personality disorders	F21, F60X
Dissociative disorders	F44X	Substance-related and addictive disorders	F10X to F19X
Elimination disorders	F 980, F981	Suicide and self-injury	R45851, T1491, T1491XA, T1491XS, T36X, T65X
Feeding and eating disorders	F500X, F982X	Sexuality and gender identity	F52X, F64X, F65X

ICD, International Classification of Diseases.

**Table 2:** Characteristics of the participants.

	Exposed Cohort ^a^ (*n* = 18,799)	Matched Unexposed Cohort ^b^ (*n* = 187,990)
Male, *n* (%)	10,684 (56.8)	106,840 (56.8)
Birth residence, *n* (%)		
Seoul	5283 (28.1)	56,554 (30.1)
Metropolitan	4300 (22.9)	40,833 (21.7)
City or Rural	8929 (47.5)	88,077 (46.9)
Missing	287 (1.5)	2526 (1.3)
Socioeconomic status, *n* (%)		
Low (<25th percentile)	4727 (25.1)	45,474 (24.2)
Intermediate (25th–75th percentile)	9166 (48.8)	92,830 (49.4)
High (>75th percentile)	4528 (24.1)	45,461 (24.2)
Missing	378	4225
Calendar year at birth, *n* (%)		
2002–2003	9456 (50.3)	94,560 (50.3)
2004–2005	9343 (47.7)	93,430 (49.7)
ICU admission within 3 months of birth, *n* (%)		
Yes	9434 (50.2)	684 (0.4)
No	9365 (49.8)	187,306 (99.6)
Any perinatal status, *n* (%) ^c^		
Yes	14,871 (76.1)	45,078 (24.0)
No	3928 (21.0)	142,912 (76.0)
Bronchopulmonary dysplasia, *n* (%)		
Yes	608 (3.2)	19 (0.01)
No	18,191 (96.8)	187,971 (99.99)
Respiratory distress syndrome, *n* (%)		
Yes	4588 (24.4)	574 (0.3)
No	14,211 (75.6)	187,416 (99.7)
Congenital heart disease, *n* (%)		
Yes	8370 (44.5)	3365 (1.8)
No	10,429 (55.5)	184,625 (98.2)
Ventilation duration (days), median (IQR)	12 (12–15)	
Duration of hospital stay (days), median (IQR)	25 (15–40)	
Age at intubation (months), median (IQR)	3 (0–21)	

ICU, intensive care unit; IQR, interquartile range. Values are expressed as number (%) or median (IQR). ^a^ The exposed cohort included individuals who underwent intubation during hospitalization within the follow-up period. ^b^ 1:10 matched unexposed cohort was selected using random matching based on sex, birth year, and intubation age. Matched unexposed cohort was assigned to the same intubation age as their exposed counterparts. In the matched unexposed cohort, follow-up continued until a mental health diagnosis or the occurrence of intubation. ^c^ Any perinatal status: disorder related to length of gestation and fetal growth; birth injury; infections specific to the perinatal period; hemorrhagic disorders of fetus and newborn; congenital malformation; and chromosomal abnormalities.

**Table 3:** Perinatal status.

	Exposed Cohort ^a^ (*n* = 18,799)	Matched Unexposed Cohort ^b^ (*n* = 187,990)
Slow fetal growth and fetal malnutrition		
Yes	330 (1.8)	226 (0.1)
No	18,469 (98.2)	187,764 (99.9)
Disorders related to short gestation		
Yes	4810 (25.6)	1215 (0.7)
No	13,989 (74.4)	186,778 (99.3)
Disorder related to long gestation		
Yes	30 (0.2)	37 (0.1)
No	18,769 (99.8)	187,953 (99.9)
Birth injury		
Yes	174 (0.9)	338 (0.2)
No	18,625 (99.1)	187,652 (99.8)
Infection specific to the perinatal period		
Yes	3774 (20.1)	11,151 (5.9)
No	15,025 (79.9)	176,839 (94.1)
Hemorrhagic disorders of perinatal period		
Yes	5009 (26.6)	13,801 (7.3)
No	13,790 (73.4)	174,188 (92.7)
Congenital malformation		
Yes	11,025 (58.7)	25,483 (13.6)
No	7774 (41.4)	162,507 (86.4)
Chromosome abnormalities		
Yes	602 (3.2)	314 (0.2)
No	18,197 (96.8)	187,676 (99.8)

Values are expressed as number (%). ^a^ The exposed cohort included individuals who underwent intubation during hospitalization within the follow-up period. ^b^ 1:10 matched unexposed cohort was selected using random matching based on sex, birth year, and intubation age. Matched unexposed cohort were assigned to the same intubation age as their exposed counterparts. In the matched unexposed cohort, follow-up continued until a mental health diagnosis or the occurrence of intubation.

**Table 4:** Stratified analysis of incidence rates and hazard ratios of mental health disorders.

	Cases/Accumulated Person-Years × 1000 (Incidence Rate/1000 Person-Years)		Hazard Ratio (95%CI)	Interaction *p*-Value
	Exposed Cohort ^a^	Matched Unexposed Cohort ^b^		
Sex				
Female	2218/84.9 (26.1)	15,580/1188.7 (13.1)	1.93 (1.80–2.07)	0.008
Male	3101/103.9 (29.9)	21,577/1493.1 (14.5)	1.74 (1.64–1.85)	
Calendar year at birth				
2002–2003	2772/99.1 (28.0)	20,696/1399.6 (14.8)	1.75 (1.65–1.86)	<0.001
2004–2005	2547/89.7 (28.4)	16,461/1282.3 (12.8)	1.92 (1.79–2.05)	
Birth residence				
Seoul	1513/52.8 (28.7)	11,201/803.8 (13.9)	1.78 (1.64–1.93)	0.436
Metropolitan	1211/44.4 (27.3)	8029/583.9 (13.8)	1.70 (1.54–1.88)	
City or Rural	2506/88.8 (28.2)	17,400/1257.9 (13.8)	1.90 (1.78–2.02)	
ICU admission within 3 months of birth				
Yes	3006/112.5 (26.7)	163/9.6 (17.0)	1.38 (1.17–1.62)	0.002
No	2313/76.3 (35.0)	36,994/2672.3 (13.8)	1.88 (1.80–1.97)	
Any perinatal status ^c^				
Yes	4643/767.6 (27.7)	10,117/626.3 (16.2)	1.55 (1.47–1.63)	<0.001
No	676/21.1 (32.0)	27,040/2055.5 (13.2)	3.06 (2.82–3.32)	
Age at intubation				
<4 month	3043/116.5 (26.1)	18,242/1378.8 (13.2)	1.34 (1.17–1.54)	<0.001
4–23 month	1280/47.1 (27.2)	12,550/892.7 (14.1)	1.47 (1.37–1.57)	
>23 month	996/25.2 (39.5)	6365/410.3 (15.5)	2.45 (2.27–2.64)	
Bronchopulmonary dysplasia				
No	37,146/2681.6 (13.9)	5049/183.0 (27.6)	1.81 (1.73–1.89)	0.006
Yes	11/0.2 (54.8)	270/5.7 (47.1)	0.71 (0.37–1.37)	
Respiratory distress syndrome				
No	3783/132.6 (28.5)	37,036/2673.9 (13.9)	1.85 (1.76–1.94)	0.535
Yes	1536/56.2 (27.3)	121/8.0 (15.2)	1.80 (1.43–2.25)	
Congenital heart disease				
No	2675/89.1 (30.0)	3604/2635.6 (13.8)	2.68 (2.52–2.85)	<0.001
Yes	2644/99.7 (26.5)	853/46.3 (18.4)	1.30 (1.19–1.42)	

ICU, intensive care unit; CI, confidence interval. Values are expressed as mental health disorder cases/accumulated 1000 person-years (incidence rate). ^a^ The exposed cohort included individuals who underwent intubation during hospitalization within the follow-up period. ^b^ 1:10 matched unexposed cohort was selected using random matching based on sex, birth year, and intubation age. Matched unexposed cohort was assigned to the same intubation age as their exposed counterparts. In the matched unexposed cohort, follow-up continued until a mental health diagnosis or the occurrence of intubation. ^c^ Any perinatal status: disorder related to length of gestation and fetal growth; birth injury; infections specific to the perinatal period; hemorrhagic disorders of fetus and newborn; congenital malformation; and chromosomal abnormalities.

**Table 5:** Dose–response analysis of mental health disorders based on hospital stay and ventilator duration.

	Number of Mental Health Disease Cases/Number of Accumulated Person-Years × 1000 (Incidence Rate/1000 Person-Years)	Adjusted Hazard Ratio (95%CI)	*p*-Value
Hospital stay ≤ 25 days			
Yes	2097/9.3 (22.6)	1.68 (1.60–1.77)	<0.001
No	3222/9.6 (33.5)	2.77 (2.62–2.93)	
Ventilator duration ≤ 12 days			
No	725/2.7 (26.8)	1.89 (1.75–2.04)	0.694
Yes	4359/15.4 (28.3)	2.02 (1.92–2.12)	

CI, confidence interval. Values are expressed as mental health disorder cases/accumulated 1000 person-years (incidence rate).

**Table 6:** Sensitivity analysis of mental health disorders by number of disorders.

Mental Health Disorders	Cases/Number of Accumulated Person-Years × 1000 (Incidence Rate/1000 Person-Years)		Adjusted Hazard Ratio (95%CI)
	Exposed Cohort ^a^	Matched Unexposed Cohort ^b^	
>2 types	2761/18.9 (146.3)	15,717/268.2 (58.6)	2.51 (2.41–2.62)
>3 types	1532/18.9 (81.2)	8329/268.2 (31.1)	2.63 (2.49–2.78)
>5 types	475/18.9 (25.2)	2391/268.2 (8.9)	2.82 (2.55–3.11)

CI, confidence interval. Values are expressed as mental health disorder cases/accumulated 1000 person-years (incidence rate). ^a^ The exposed cohort included individuals who underwent intubation during hospitalization within the follow-up period. ^b^ 1:10 matched unexposed cohort was selected using random matching based on sex, birth year, and intubation age. Matched unexposed cohort was assigned to the same intubation age as their exposed counterparts. In the matched unexposed cohort, follow-up continued until a mental health diagnosis or the occurrence of intubation.

**Table 7:** Characteristics before and after IPTW for sensitivity analysis.

	Cohort Before IPTW			Cohort After IPTW		
	Exposed Cohort	Matched Unexposed Cohort	Standardized Mean Difference, %	Exposed Cohort	Matched Unexposed Cohort	Standardized Mean Difference, %
Participant, *n*	18,799	187,990		27,574.29	1,799,364	
Male	10,684 (56.8)	106,840 (56.8)	0	15,636 (57)	900,652 (50)	8.6
Birth residence						
Seoul	5283 (28.1)	56,554 (30.1)	4.4	7863 (29)	540,391 (30)	1.3
Metropolitan	4300 (22.9)	40,833 (21.7)	2.8	6321 (23)	390,769 (22)	0.1
City or Rural	8929 (47.5)	88,077 (46.9)	1.3	13,100 (48)	844,283 (47)	0.9
Socioeconomic status						
Low (<25th percentile)	4727 (25.1)	45,474 (24.2)	2.2	6923 (25)	435,135 (24)	0.7
Intermediate (25th–75th percentile)	9166 (48.8)	92,830 (49.4)	1.3	13,584 (49)	893,067 (50)	1.9
High (>75th percentile)	4528 (24.1)	45,461 (24.2)	0.2	6688 (24)	431,647 (24)	1.8
Calendar year at birth						
2002–2003	9456 (50.3)	94,560 (50.3)	0	13,939 (51)	932,104 (52)	1.2
2004–2005	9343 (47.7)	93,430 (49.7)		13,636 (49)	867,260 (48)	
ICU admission within 3 months of birth						
Yes	9434 (50.2)	684 (0.4)	139.9	13,171 (48.2)	8931 (0.5)	14.1
No	9365 (49.8)	187,306 (99.6)		14,403.3 (52)	1,790,433 (100)	
Slow fetal growth and fetal malnutrition						
Yes	330 (1.8)	226 (0.1)	17	464 (2)	2388 (0.13)	5.3
No	18,469 (98.2)	187,764 (99.9)		27,110 (98)	1,796,976 (99.87)	
Disorders related to short gestation						
Yes	4810 (25.6)	1215 (0.7)	79.5	6535 (24)	12,743 (0.71)	13
No	13,989 (74.4)	186,778 (99.3)		21,040 (76)	1,786,621 (99.29)	
Disorder related to long gestation						
Yes	30 (0.2)	37 (0.1)	4.7	42 (0)	393 (0.02)	0.8
No	18,769 (99.8)	187,953 (99.9)		27,531 (100)	1,798,971 (99.98)	

IPTW, inverse probability of treatment weighting; ICU, intensive care unit. Values are expressed as number (%).

## Data Availability

This study is based on data from the NHIS of the Republic of Korea. Applications for NHIS data use are reviewed by the Inquiry Committee of Research Support, and if approved, raw data are provided to applicants for a fee. We cannot directly share the data, analytical methods, or research materials because of the intellectual property rights held by NHIS. Nonetheless, investigators wishing to reproduce our results or replicate the procedures may apply to access the database, which is available for research purposes.

## Abbreviations

The following abbreviations are used in this manuscript:

ASD	Autism Spectrum Disorder
ADHD	Attention-Deficit/Hyperactivity Disorder
NHIS	National Health Insurance Service
ICD	International Classification of Diseases
DSM	Diagnostic and Statistical Manual of Mental Disorders
ICU	Intensive Care Unit
BPD	Bronchopulmonary Dysplasia
CHD	Congenital Heart Disease
IQR	Interquartile Range
HR	Hazard Ratio
CI	Confidence Interval
IPTW	Inverse Probability of Treatment Weighting
